# Stepwise work hardening induced by individual grain boundary in Cu bicrystal micropillars

**DOI:** 10.1038/srep15631

**Published:** 2015-10-22

**Authors:** L. L. Li, Z. J. Zhang, J. Tan, C. B. Jiang, R. T. Qu, P. Zhang, J. B. Yang, Z. F. Zhang

**Affiliations:** 1 Shenyang National Laboratory for Materials Science, Institute of Metal Research, Chinese Academy of Sciences, 72 Wenhua Road, Shenyang 110016, P.R. China

## Abstract

Vast experiments have demonstrated that the external specimen size makes a large difference in the deformation behavior of crystalline materials. However, as one important kind of internal planar defects, the role of grain boundary (GB) in small scales needs to be clarified in light of the scarce and inconsistent experimental results at present. Through compression of Cu bicrystal and its counterpart monocrystal micropillars, it is found that, in contrast to the monocrystals, the bicrystals are characterized by work hardening with discrete strain bursts. Interestingly, the stress rise between two adjacent strain bursts of the bicrystals increases with the decrease of specimen size. The results suggest that GBs play a critical role in the work hardening of materials in small scales, which may provide important implications to further understand the general work hardening behaviors of materials in the future.

Both the internal sizes of the microstructural units and the external sizes of the specimens govern the mechanical properties of materials in micro and smaller scales simultaneously^1^,^2^,^3^. In recent years, materials in small scales have been investigated a lot by applying micromechanical tests to the specimens fabricated by focus ion beams (FIB) milling method^2^. Investigations on monocrystal pillars reveal that the strength increases with the decrease of the sample size due to the geometrical constraints on the dislocation nucleation and motion^4^,^5^,^6^. In addition, the deformation in small scales is characterized by discrete strain burst^2^,^7^ and low hardening rate even up to large strains^2^,^8^. Generally speaking, conventional strain hardening in bulk materials is invalid in the specimen with limited volumes since the dislocation density does not grow significantly with strain^9^.

Grain boundary (GB) is an important kind of internal planar defects in crystalline materials. By working as sinks, sources or obstacles to lattice dislocations, the GBs always play a crucial role in the mechanical properties of polycrystalline metals^10^. The GBs are opaque to dislocation motion and it was proposed by Hall and Petch that decreasing grain sizes would effectively increase the yield strength of materials^11^,^12^. Moreover, the GBs could improve the initial work hardening behavior of bulk materials by providing back stress to dislocations^13^,^14^,^15^. On the other hand, the GB-mediated deformation in the way of grain rotation and GB sliding can become prominent when the average grain size is below some critical value^16^,^17^. Beyond bulk materials, the role of GBs in small scales has also been investigated via nanocrystal, multi-crystal or bicrystal pillars in micro or nano scale in recent years^7^,^18^,^19^,^20^,^21^,^22^. However, there is large scatter of the experimental results of pillars with GBs under compression. The strength of nanocrystal pillars may increase or decrease as a function of the extrinsic specimen size^19^,^22^. Wherein, the weakening effect is likely due to the activation of GB-mediated processes^22^; instead, the strength significantly rises when twin boundaries (TBs) are present to greatly impede dislocation glide^19^.

In the previous studies, polycrystalline aggregates were mainly employed and the existence of numerous GBs makes it difficult to distinguish the intrinsic role of individual boundary. To overcome this drawback, bicrystals with an individual GB were employed. Direct evidence has been offered to show that high-angle GBs have low resistance to fatigue cracking and serve as the preferential fatigue cracking sites^23^, while the TBs have variable and tunable fatigue cracking behaviors^24^. However, the GB effect is not remarkable in tension or compression behaviors of bulk Cu bicrystals as a result of the quite low GB fraction. In micro or smaller scales, the fraction is so high that the GBs can strongly influence the deformation behaviors of the bicrystals by interacting with lattice dislocations. Earlier studies indicated that the GB could work as a dislocation trap to induce strengthening or smoothening effects of the bicrystal micropillars^18^,^20^,^21^. On the contrary, the GB was reported to work as a dislocation sink to contribute to the intermittent strain bursts and decreasing the work hardening degree of Al bicrystal nanopillars^7^. The divergences might be derived from different GB characters^25^,^26^, inclination angles^27^ or specimen sizes^7^,^21^. It could be seen that GB’s role in the deformation behaviors of the bicrystal pillars in small scales has not been thoroughly understood, including the work hardening behaviors, which needs to be further clarified with more studies.

Micro-compression tests were deliberately designed in this investigation with a series of Cu bicrystal and the two counterpart monocrystal micropillars. Here we present that the bicrystals deform more homogeneously than the monocrystals and are characterized by stepwise work hardening behavior. Moreover, it is interesting to find that the stress increase between two adjacent strain bursts of the bicrystals is inversely proportional to the specimen size. The GB could provide restrict to the annihilation and operation of dislocations which gives rise to the specific deformation and work hardening behaviors of the bicrystal micropillars.

## Results

**Figure 1:**
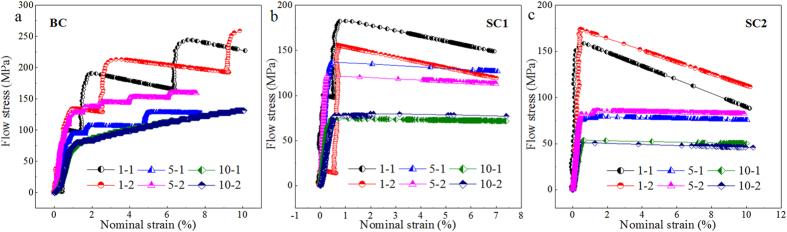
Compression curves of micropillars. Compression curves of (**a**) bicrystal (**b**) monocrystal (SC1) and (**c**) monocrystal (SC2) pillars with different sizes (1 μm, 5 μm and 10 μm).

The two monocrystal pillars were named as SC1 and SC2 respectively, while the bicrystal pillar was named as BC. The crystallographic orientations of SC1 and SC2 are <345> and <136>, respectively. Representative compressive stress-strain curves for monocrystal and bicrystal micropillars with the sizes of 1 μm, 5 μm and 10 μm are displayed in Fig. 1. Obviously, all these micropillars are much stronger than their bulk counterparts. Besides, the smaller the specimen size is, the larger scatter the datum shows. As to the monocrystal pillars, the flow stress increases as the specimen size decreases, indicating a strong sample size effect analogous to the former studies^28^,^29^. The yield stresses of the two monocrystal pillars are different ascribed to their different loading directions^30^. During the compression, monocrystal pillars exhibited a short period of elastic loading firstly and a catastrophic plastic flow event with a little stress drop subsequently. The instable plastic flow continued for a long strain regime which may be closely concerned with the avalanche-like motion of dislocations^31^. Moreover, there is no work hardening of monocrystal pillars similar to the former works^2^,^4^,^32^.

**Figure 2:**
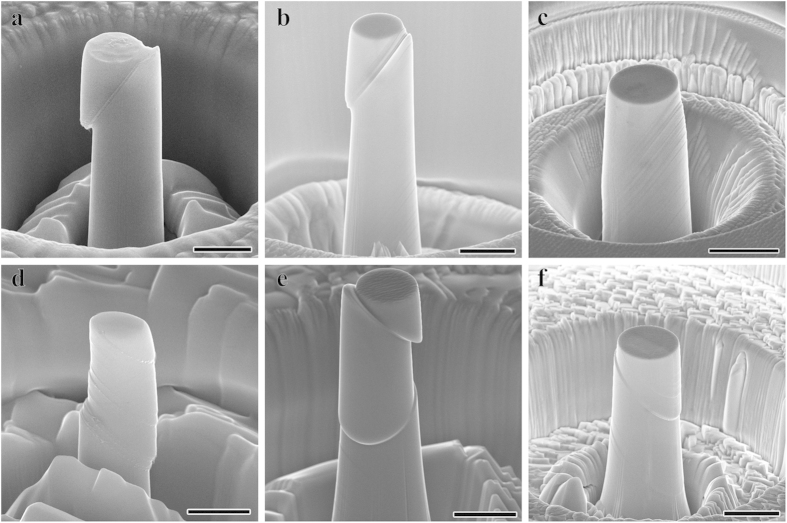
Slip morphologies of the compressed monocrystal pillars with different sizes. Slip morphologies of the SC1 pillars of (**a**) 1 μm, (**b**) 5 μm and (**c**) 10 μm in diameter, respectively. The scale bar is 1 μm, 5 μm and 10 μm, respectively. Slip morphologies of SC2 pillars of (**d**) 1 μm, (**e**) 5 μm and (**f**) 10 μm in diameter, respectively. The scale bar is 1 μm, 5 μm and 1 μm, respectively.

The stress-strain curves are correlated with the characteristic deformation morphologies. Surface observations of the deformed monocrystal micropillars with different diameters are displayed in Fig. 2. Wherein, Fig. 2a–c represent the SC1 monocrystals, while Fig. 2d–f represent the SC2 monocrystal pillars, respectively. In general, strongly localized slip bands (SBs) with significant shear offsets are prevalent on the sample surface since the dislocations could easily escape from the crystals to the free surface^2^,^32^. Different slip systems operated in the two monocrystal pillars attributed to their different orientations. In addition, as shown in Fig. 2e, a secondary slip system operated in the SC2 monocrystal pillars, which might be related to the fact that the its Schmid factor is close to the highest one. In addition, multiple slip systems could be activated in micro scale^8^,^32^ and simulations show that the dominance of the maximum Schmid-factor system drops as the specimen size decreases^33^,^34^. Although both the primary and secondary SBs were activated in the SC2 monocrystal pillars, they had no intersection with each other so as not to facilitate the work hardening behavior.

For the bicrystal pillars, however, they exhibited distinct features from the monocrystal pillars in both the stress-strain curves and deformation morphologies. It could be seen from Fig. 1a that the flow stress also increases in a “smaller is stronger” fashion for the bicrystal micropillars as observed in the nano and micro bicrystal pillars before^7^,^18^. Different from the two monocrystal pillars, the stress-strain curves of the bicrystals are composed of many regular strain bursts which are separated by elastic loading segments. Each strain burst continued at nearly the same stress level and the subsequent strain burst occurred at a higher stress level. That is, the flow stress of the bicrystals increases in a stepped mode exhibiting a distinct work hardening feature.

**Figure 3:**
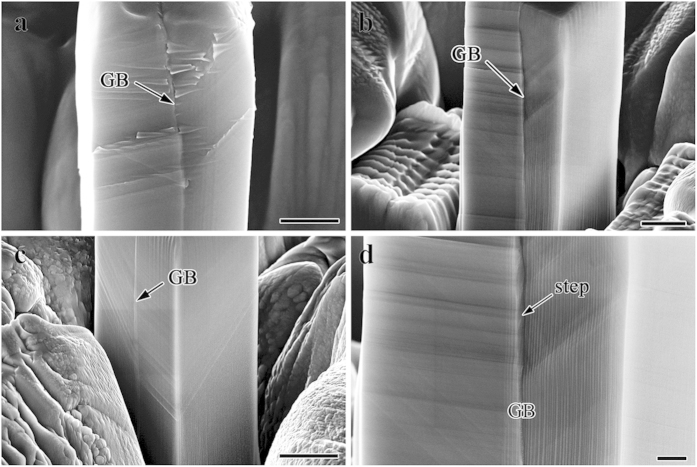
Slip morphologies of the compressed bicrystal pillars with different sizes. Slip morphology of the BC pillars with the sizes of (**a**) 1 μm, (**b**) 5 μm and (**c**) 10 μm, respectively. The scale bar is 500 nm, 2 μm and 5 μm, respectively. (**d**) Enlarged image of local position from Fig. 3b. Scale bar, 500 nm.

The representative deformation morphologies of the bicrystal micropillars are displayed in Fig. 3. Figure 3a–c demonstrate BC pillars with sizes of about 1 μm, 5 μm and 10 μm, respectively. Different SBs operated in the two component grains due to the different grain orientations and all of them ceased at the GB. Besides, there are also some secondary SBs showed up due to the strain or stress incompatibility^35^ and intersected with the primary SBs as shown in Fig. 3a. The secondary SBs usually arise in the vicinity of the GB in bulk materials, nevertheless, the SBs distributed through the entire grain on one side of the GB due to the size limit at present. Comparatively speaking, the deformation of the bicrystal pillars is undertaken by SBs which distributed more homogeneously and widely than those in monocrystal pillars. The height of the slip steps on the surface of bicrystal pillars is obviously lower than that of monocrystal pillars. In addition, Fig. 3d is an enlarged image of local position from Fig. 3b. It could be observed that there is a shear step formed along the GB which may arise from the deformation incompatibility between the two component grains. It has been proposed that frictional sliding along the GB plane served as the dominant deformation mechanism in Al nano-bicrystals^27^. There might also be little GB sliding in the present bicrystals since there should be resolved shear stress along the present GB which is not perfectly parallel to the loading direction. More studies on the investigation of the GB sliding behavior are still underway.

## Discussion

**Figure 4:**
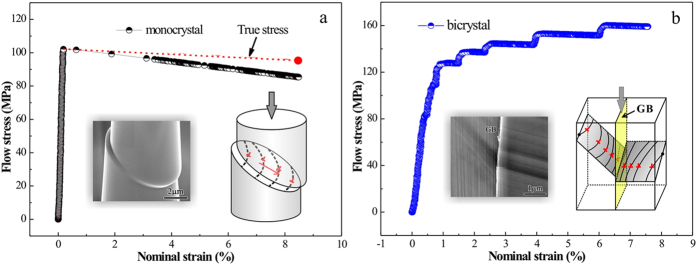
Schematics of stress-strain curves and dislocation behaviors for monocrystal and bicrystal pillars. (**a**) The engineering stress (black line) and true stress (red line) amended by the actual stressed area of monocrystal pillars. The inset at the left bottom is the actual deformation morphology while the one at the right bottom is the sketch of the dislocation behavior. (**b**) Representative stress-strain curves for bicrystal pillars. The inset at the left bottom is the actual deformation morphology while the one at the right bottom is the sketch of the dislocation behavior.

Based on the above results, the two monocrystal pillars show stress-strain curves of the same characteristics without work hardening. During the compression, the stressed area of monocrystal pillar decreases with the compressive strain since the deformation is highly localized in the SBs, as illustrated by the insets in Fig. 4a. Based on the scanning electron microscope (SEM) observation, the actual stressed area was measured and the flow stress was amended. The flow stress increased a little, but not enough to appear as work hardening in the present strain range. As shown in Fig. 4b, the true stress of the bicrystal pillars can be considered as equal to the engineering stress in the present strain range, since the stressed area did not change much due to the homogeneous deformation. Compared with monocrystal pillars, there is definite work hardening behavior of bicrystal pillars.

The value of macroscopic shear strain *γ* is related to the density of mobile dislocations *ρ* and their travel distance 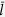 with the Orowan’s equation^36^: 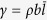. For monocrystal pillars, due to the low initial dislocation density^9^,^37^, high stress is required to nucleate sufficient dislocations to sustain the shear strain^38^,^39^. It is the same case in the bicrystal pillars and hence the yield strength is also higher than bulk counterparts due to the low initial dislocation density in the limited specimen size. In the following deformation process, different dislocation behaviors occur in the monocrystals and bicrystals. The deformed bicrystal and monocrystal pillars with the size of 5 μm were milled into longitudinal thin-foil sections for transmission electron microscopy (TEM) examination.

**Figure 5:**
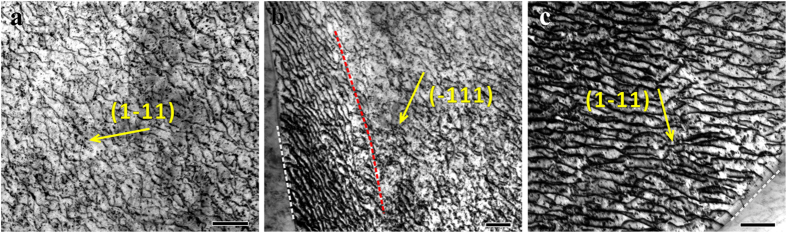
Dislocation configurations of the compressed monocrystal and bicrystal pillars with the size of 5 μm. (**a**) Bright-field TEM image with 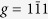 near the [110] pole of the deformed [316]-oriented monocrystal; (**b**) bright-field TEM image with g = 111 near the 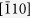 pole of the [316]-oriented grain and (**c**) bright-field TEM image with 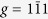 near the 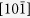 pole of the [345]-oriented grain of the deformed bicrystal. The white dashed lines indicate the GB and the scale bar is 200 nm.

The dislocation configuration of the <136> monocrystal pillar with the nominal strain of ~8.4% is shown in Fig. 5a while the dislocation configurations in the two component grains of the bicrystal pillars with the nominal strain of ~4.7% are exhibited in Fig. 5b,c. The Burgers vector of the dislocations in Fig. 5c was determined to be 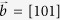 which belongs to the primary slip system with the highest Schmid factors. Meanwhile, the Burgers vectors of the dislocations in Fig. 5a,b were determined to be 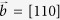 which belongs to the secondary slip systems indicating that the Schmid’s law may break down in small scale^33^,^34^. In general, the dislocation density should be higher with higher strain. However, the dislocation density of the compressed bicrystal is a little higher than that in the monocrystal, while they are estimated to be in the same order of 10^14^/m^2^. Wherein, the dislocation density was measured by the line-intercept method^8^,^40^. Therefore, it could be deduced that the dislocations can readily zip through the monocrystal specimen and annihilate at the surface leading to the low dislocation density. It has been revealed previously that the dislocation density of the monocrystal micropillars did not increase significantly after serious deformation by *in-situ* and *ex-situ* TEM investigations^5^,^9^. Thus, in the monocrystal pillars, dislocations have low chance to interact with each other^5^,^9^,^41^ and thus work hardening is absent as shown by the sketch map in Fig. 4a.

On the other hand, one end of some dislocations is usually pinned by the GB as shown in Fig. 5b,c. That is, the GB can provide obstruction to dislocation annihilation which leads to the global higher dislocation density than monocrystal. Additionally, take the red line in Fig. 5b as a dividing line, the dislocations distribute more densely when approaching to the GB which implies that the dislocations could pile up at the GB. During deformation, mobile dislocations could pile up at GBs or annihilate at surface, so new dislocations should be nucleated and emitted to sustain the following plastic deformation. As reflected by the stress-strain curves in Fig. 4b, during each strain burst, the activated dislocations slip through the grain and then are blocked by the GB leading to exhaustion of mobile dislocations. Afterwards, stress rise is needed to activate new dislocations^38^,^41^,^42^ to produce the subsequent strain burst which provides the major contribution to the work hardening behavior of bicrystal pillars.

**Figure 6:**
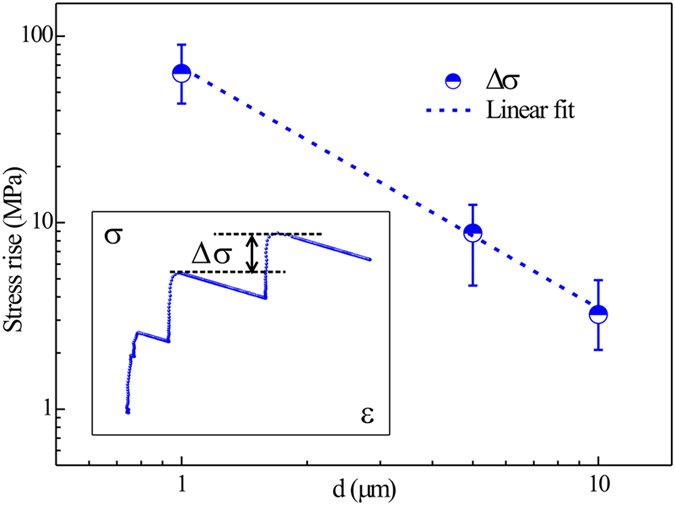
Statistics of the stress rise amplitude with the size of the bicrystal pillars. The stress rise (Δσ) between two strain bursts increases with the decrease of the bicrystal size. Sketch for Δσ is shown as the inset.

The amplitudes of the stress rise Δσ (illustrated by the inset in Fig. 6) for the bicrystal micropillars are collected and analyzed. It is found that Δσ is very concentrated for the bicrystal pillars with the same size. Then the amplitudes of stress rise for the bicrystal pillars with all the sizes (1 μm, 5 μm and 10 μm) are collected and listed in Fig. 6. It is clear that the stress rise amplitude Δσ is proportional to *d*^−1.29^, exhibiting a strong size effect. As shown by the stress-strain curves in Fig. 1, differences exist between the yield stresses of the two monocrystal pillars with the same size, which are estimated to be 24.5 ± 0.3 MPa for the 10 μm pillars, 49.8 ± 5.5 MPa for the 5 μm pillars and 4.3 ± 7.7 MPa for the 1 μm pillars. Obviously, yield stresses scatter largely when the pillar size is reduced to 1 μm. However, these differences in the yield strength are quite different from the corresponding Δσ, which are about 3.2 MPa, 8.8 MPa and 63.2 MPa for 10 μm, 5 μm and 1 μm pillars, respectively. Therefore, Δσ is more closely related to the existence of GB than to the orientation differences between the two monocrystal counterparts.

In bulk polycrystals, the dislocations piled up or stored at the GB will contribute to the building up of back stresses^13^,^15^. At low strain level, the strengthening effect of GB increases evidently with decreasing the grain size^13^,^14^. It is conceivable that the GB back stress *τ* exerted on a source could be one reason for the dislocation immobilization. The back stress *τ* could be calculated by^43^:


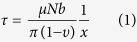


where in, *ν* is Poisson’s ratio, *μ* is shear modulus, *b* is the magnitude of the Burgers vector. The separation *x* between source and GB should be proportional to the specimen size^44^ and it is assumed to be about half of the pillar’s size. The back stress exerted on each dislocation in the pilling-up, i.e., the stress rise Δτ′ needed to release one dislocation in the pilling-up can be estimated from Eq. (1) as below:


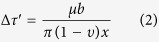


It is obvious that both Δτ′ and Δσ increase with the decrease of the specimen size, but in different ways: Δτ′ is proportional to *d*^−1^ while Δσ is proportional to *d*^−1.29^. The discrepancy may arise from the rough estimation in the separation distance *x* and the specific number of dislocations released from the source for one strain burst, both of which are hard to determine and in need of further study. Even so, it confirms that the GB poses barrier both to the dislocation motion and source reemission which facilitates the work hardening behavior of the bicrystal pillars, which could be extended to bicrystal pillars with other kind of GBs.

In conventional bulk materials, there are numerous dislocation sources and dislocations can multiply easily which smoothes the plastic deformation. Thus stress rise is principally associated with the strong interactions between dislocations, leading to the typical work hardening behavior. In large scales, the initial work hardening behavior in polycrystals is dependent on the grain size owing to the GB back stress to the dislocation motion. However, in small scales, changes take place in nucleation and multiplication of the dislocations, which would then influence the dislocation interactions with the GB. In the bicrystal micropillars, the deformation was controlled by progressive activation and subsequent exhaustion of dislocations, both of which are associated with the GB. To be specific, the GB not only traps the mobile dislocations but also generates back stress to other dislocations. Therefore, it is understandable that the work hardening behavior of bicrystal micropillars exhibit as discrete strain bursts during compression, which is distinct from the smooth work hardening of bulk materials.

To summarize, the present study reveals different deformation behaviors of Cu monocrystal and bicrystal pillars in the micro scale. In contrary to the uninhibited plastic flow without work hardening in the monocrystal pillars, the bicrystal pillars exhibit stepwise work hardening behavior with discrete strain bursts. In particular, the stress rise amplitude is inversely proportional to the specimen size. It is suggested that the GB obstruction to dislocation annihilation stabilizes the deformation while the GB barrier effect to dislocation motion alleviates the softening of the pillars. Both the GB and the sample size influence the deformation and work hardening behaviors of the bicrystal pillars. The present results may provide important implications to further understand the general work hardening behaviors of materials in the future.

## Materials and Experiments

The bulk Cu bicrystal with a high-angle GB was grown from 5N purity Cu by the Bridgman method. Micropillars of the bicrystal and two counterpart monocrystals were prepared from the bulk bicrystal by focused ion beam (FIB) milling. Wherein, the GB is parallel to the loading axis. The orientations of two monocrystals are <345> and <136> respectively and the misorientation of the GB is about 40° as determined by the electron backscatter diffraction (EBSD) method. A cuboid sample with the size of 8 mm × 8 mm × 2 mm was carefully mechanically ground and electro-polished firstly as a base and then micropillar specimens were fabricated from this bulk sample. The FIB milling was carried out in a Nova 200 Nanolab dual beam system at 30 kV with gallium as the ion source. For coarse milling, a 3 nA ion-beam current was used and the current was gradually stepped down to 30 pA for the final polishing analogous to the method used before^9^. A series of cylindrical monocrystal pillars were produced with the diameters ranging from ~1 μm to ~10 μm and the aspect ratio ranging from 5:1 to 7:1. The pillars were slightly tapered within 2° and the sample sizes were taken from the head of the pillars. Bicrystal pillars with nearly square-shaped cross section were produced with the nominal width ranging from ~1 μm to ~10 μm and the aspect ratio ranging from 3:1 to 4:1. Uniaxial compression tests were conducted using a Nanoindenter G200 (Agilent Technologies) equipped with a flat punch. The nominal strain rate ranged from 3 ~ 5 × 10^−4^ for all the specimens. In addition, comprehensive SEM observations of the surface morphologies before and after compression were carried out via Supra 55 SEM with the specimens at 52° tilt. Deformed bicrystal and monocrystal pillars with the size of 5 μm were milled with FIB along the loading direction into thin-foil sections for *ex-situ* TEM examination of the dislocation structures. These deformed pillars were first coated with a platinum protective layer in the FEI Helios 650 Dual beam FIB system, and then parts of pillars containing slip traces were cut off from the base, finally they were transferred by an *in-situ* lift-out system (OmniProbe^TM^ 200) to a Cu grid. The sample was first milling by Ga+ ions beam at an accelerating voltage of 30 kV and was thinned to 1 ~ 2 μm with a beam current of 0.77 nA. Afterwards, a beam current of 33 pA was employed to further thin the sample into electron transparency. The final size of the TEM specimen is about ~6 μm × ~ 6 μm with the thickness of 60 ~ 80 nm. All TEM observations were performed on a 200 kV FEI Tecnai F20 fitted with a field-emission electron source.

## Additional Information

**How to cite this article**: Li, L. L. *et al.* Stepwise work hardening induced by individual grain boundary in Cu bicrystal micropillars. *Sci. Rep.* **5**, 15631; doi: 10.1038/srep15631 (2015).
